# Supplementary material for: Effectiveness of Virtual Reality and Feedback to Improve Gait and Balance in Patients with Diabetic Peripheral Neuropathies: Systematic Review and Meta-Analysis
Source: Healthcare (Basel). 2023 Nov 24;11(23):3037. doi: 10.3390/healthcare11233037 (PMC10706033; doi:10.3390/healthcare11233037)
Supplement: Supplementary file 1 [file healthcare-11-03037-s001.zip › healthcare-2696305-supplementary.pdf]

List S1: Excluded articles and reasons.

EXCLUDED ARTICLES BASED ON EXCLUSION CRITERIA (n = 60)

No diabetes (n = 5)

Fukuda, K.; Kanazawa, H.; Aizawa, Y.; Ardell, J.L.; Shivkumar, K. Cardiac Innervation and Sudden Cardiac Death. *Circ Res* **2015**, *116*, 2005–2019.

Femery, V.G.; Moretto, P.G.; Hespel, J.-M.G.; Thévenon, A.; Lensel, G. A Real-Time Plantar Pressure Feedback Device for Foot Unloading. *Arch Phys Med Rehabil* **2004**, *85*, 1724–1728.

Klein, R.M.; Mann, R.J.; Walling, A.D. A Thematic Approach to Enhance Clinical Content in a Cell and Tissue Biology Course. *Academic Medicine* **2002**, *77*, 1173–1174.

Pinto, M.V.; Barreira, A.A.; Bulle, A.S.; de Freitas, M.R.G.; França, M.C.; Gondim, F.A.A.; Marrone, C.D.; Marques, W.; Nascimento, O.J.M.; Rotta, F.T.; et al. Brazilian Consensus for Diagnosis, Management and Treatment of Transthyretin Familial Amyloid Polyneuropathy | Consenso Brasileiro Para o Diagnóstico, Manejo e Tratamento Da Polineuropatia Amiloidótica Familiar Associada à Transtirretina. *Arq Neuropsiquiatr* **2018**, *76*, 609–621.

Templeton, C.A.; Strzalkowski, N.D.J.; Galvin, P.; Bent, L.R. Cutaneous Sensitivity in Unilateral Trans-Tibial Amputees. *PLoS One* **2018**, *13*.

No treatment (n = 26)

Cavanagh, P.R.; Simoneau, G.G.; Ulbrecht, J.S. Ulceration, Unsteadiness, and Uncertainty: The Biomechanical Consequences of Diabetes Mellitus. *J Biomech* **1993**, *26*.

Demarin, V.; Bašić-Kes, V.; Zavoreo, I.; Bosnar-Puretić, M.; Rotim, K.; Lupret, V.; Perić, M.; Ivanec, Ž.; Fumić, L.; Lušić, I.; et al. Ad Hoc Committee of the Croatian Society for Neurovascular Disorders, Croatian Medical Association: Recommendations for Neuropathic Pain Treatment. *Acta Clin Croat* **2008**, *47*, 181–191.

Dingwell, J.B.; Cusumano, J.P. Nonlinear Time Series Analysis of Normal and Pathological Human Walking. *Chaos* **2000**, *10*, 848–863.

Dingwell, J.B.; Kang, H.G.; Marin, L.C. The Effects of Sensory Loss and Walking Speed on the Orbital Dynamic Stability of Human Walking. *J Biomech* **2007**, *40*, 1723–1730.

Ettinger, L.R.; Boucher, A.; Simonovich, E. Patients with Type 2 Diabetes Demonstrate Proprioceptive Deficit in the Knee. *World J Diabetes* **2018**, *9*, 59–65.

Gomes, A.A.; Onodera, A.N.; Otuzi, M.E.I.; Pripas, D.; Mezzarane, R.A.; Sacco, I.C.N. Electromyography and Kinematic Changes of Gait Cycle at Different Cadences in Diabetic Neuropathic Individuals. *Muscle Nerve* **2011**, *44*, 258–268.

Grewal, G.; Sayeed, R.; Yeschek, S.; Menzies, R.A.; Talal, T.K.; Lavery, L.A.; Armstrong, D.G.; Najafi, B. Virtualizing the Assessment: A Novel Pragmatic Paradigm to Evaluate Lower Extremity Joint Perception in Diabetes. *Gerontology* **2012**, *58*, 463–471.

Grewal, G.S.; Bharara, M.; Menzies, R.; Talal, T.K.; Armstrong, D.; Najafi, B. Diabetic Peripheral Neuropathy and Gait: Does Footwear Modify This Association? *J Diabetes Sci Technol* **2013**, *7*, 1138–1146.

Grote, C.W.; Wilson, N.M.; Katz, N.K.; Guilford, B.L.; Ryals, J.M.; Novikova, L.; Stehno-Bittel, L.; Wright, D.E. Deletion of the Insulin Receptor in Sensory Neurons Increases Pancreatic Insulin Levels. *Exp Neurol* **2018**, *305*, 97–107.

Höhne, A.; Ali, S.; Stark, C.; Bruggemann, G.P. Reduced Plantar Cutaneous Sensation Modifies Gait Dynamics, Lower-Limb Kinematics and Muscle Activity during Walking. *Eur J Appl Physiol* **2012**, *112*, 3829–3838.

Horak, F.B.; Dickstein, R.; Peterka, R.J. Diabetic Neuropathy and Surface Sway-Referencing Disrupt Somatosensory Information for Postural Stability in Stance. *Somatosens Mot Res* **2002**, *19*, 316–326.

Khalaf, K.; Al-Angari, H.M.; Khandoker, A.H.; Lee, S.; Almahmeed, W.; Al Safar, H.S.; Jelinek, H.F. Gait Alterations in the UAE Population with and without Diabetic Complications Using Both Traditional and Entropy Measures. *Gait Posture* **2017**, *58*, 72–77.

Lin, S.I.; Chen, Y.R.; Liao, C.F.; Chou, C.W. Association between Sensorimotor Function and Forward Reach in Patients with Diabetes. *Gait Posture* **2010**, *32*, 581–585.

Mengarelli A, Tigrini A, Verdini F, Rabini RA, Fioretti S. Multiscale Fuzzy. Entropy Analysis of Balance: Evidences of Scale-Dependent Dynamics on Diabetic Patients With and Without Neuropathy. *IEEE Trans Neural Syst Rehabil Eng*. **2023**;PP. doi: 10.1109/TNSRE.2023.3248322. Epub ahead of print. PMID: 37027606.

Menz, H.B.; Lord, S.R.; Fitzpatrick, R.C. A Tactile Stimulus Applied to the Leg Improves Postural Stability in Young, Old and Neuropathic Subjects. *Neurosci Lett* **2006**, *406*, 23–26.

Morley R.E., Jr.; Richter, E.J.; Klaesner, J.W.; Maluf, K.S.; Mueller, M.J. In-Shoe Multisensory Data Acquisition System. *IEEE Trans Biomed Eng* **2001**, *48*, 815–819.

Muller, K.A.; Ryals, J.M.; Feldman, E.L.; Wright, D.E. Abnormal Muscle Spindle Innervation and Large-Fiber Neuropathy in Diabetic Mice. *Diabetes* **2008**, *57*, 1693–1701.

Palma, F.H.; Antigual, D.U.; Martínez, S.F.; Monrroy, M.A.; Gajardo, R.E. Static Balance in Patients Presenting Diabetes Mellitus Type 2 with and without Diabetic Polyneuropathy. *Arq Bras Endocrinol Metabol* **2013**, *57*, 722–726.

Suda, E.Y.; Matias, A.B.; Bus, S.A.; Sacco, I.C.N. Impact of Diabetic Neuropathy Severity on Foot Clearance Complexity and Variability during Walking. *Gait Posture* **2019**, *74*, 194–199.

Toosizadeh, N.; Mohler, J.; Armstrong, D.G.; Talal, T.K.; Najafi, B. The Influence of Diabetic Peripheral Neuropathy on Local Postural Muscle and Central Sensory Feedback Balance Control. *PLoS One* **2015**, *10*.

Van Deursen, R.W.M.; Sanchez, M.M.; Ulbrecht, J.S.; Cavanagh, P.R. The Role of Muscle Spindles in Ankle Movement Perception in Human Subjects with Diabetic Neuropathy. *Exp Brain Res* **1998**, *120*, 1–8.

van Schie, C.H.M. Neuropathy: Mobility and Quality of Life. *Diabetes Metab Res Rev* **2008**, *24*.

Vargas Matamala, M.; Tapia, C.; Sagüez, F.S.; Guerrero-Henriquez, J. Postural Performance Assessment in Aging People with Diabetes and Diabetic Peripheral Neuropathy Using a Wii Balance Board. *Disabil Rehabil*. **2023**,45(7):1202-1207. doi: 10.1080/09638288.2022.2055168.

Walker, S.C.; Helm, P.A.; Lavery, L.A. Gait Pattern Alteration by Functional Sensory Substitution in Healthy Subjects and in Diabetic Subjects with Peripheral Neuropathy. *Arch Phys Med Rehabil* **1997**, *78*, 853–856.

Yavuz, M.; Ocak, H.; Hetherington, V.J.; Davis, B.L. Prediction of Plantar Shear Stress Distribution by Artificial Intelligence Methods. *J Biomech Eng* **2009**, *131*.

Zhang, J.; Zhang, K.; Feng, J.; Small, M. Rhythmic Dynamics and Synchronization via Dimensionality Reduction: Application to Human Gait. *PLoS Comput Biol* **2010**, *6*.

No clinical trials (n = 12)

Ramdharry, G. Peripheral Nerve Disease. In *Handbook of Clinical Neurology*; **2018**; Vol. 159, pp. 403–415.

17H Congress of Advances in Physiotherapy and Rehabilitation. *Fizyoterapi Rehabilitasyon* 2018 29:2

Alam, U.; Riley, D.R.; Jugdey, R.S.; Azmi, S.; Rajbhandari, S.; D'Août, K.; Malik, R.A. Diabetic Neuropathy and Gait: A Review. *Diabetes Therapy* **2017**, *8*, 1253–1264.

Castelnuovo G., Giusti E.M., Manzoni G.M., Saviola D., Gabrielli S., Lacerenza M., et al. What Is the Role of the Placebo Effect for Pain Relief in Neurorehabilitation? Clinical Implications From the Italian Consensus Conference on Pain in Neurorehabilitation. *Front Neurol.* **2018**, 9:310. doi: 10.3389/fneur.2018.00310.

Chatwin, K.E.; Abbott, C.A.; Boulton, A.J.M.; Bowling, F.L.; Reeves, N.D. The Role of Foot Pressure Measurement in the Prediction and Prevention of Diabetic Foot Ulceration—A Comprehensive Review. *Diabetes Metab Res Rev* **2020**, *36*.

Crews, R.T.; Yalla, S.V.; Fleischer, A.E.; Wu, S.C. A Growing Troubling Triad: Diabetes, Aging, and Falls. *J Aging Res* **2013**, *2013*.

Ferris, J.K.; Timothy Inglis, J.; Madden, K.M.; Boyd, L.A. Brain and Body: A Review of Central Nervous System Contributions to Movement Impairments in Diabetes. *Diabetes* **2020**, *69*, 3–11.

Harati, Y. Diabetic Neuropathies: Unanswered Questions. *Neurol Clin* **2007**, *25*, 303–317.

Horstink, K.A.; Henricus, L.; Van Der Woude, V.; Juha, &; Hijmans, M. Effects of Offloading Devices on Static and Dynamic Balance in Patients with Diabetic Peripheral Neuropathy: A Systematic Review. *Rev Endocr Metab Disord*. **2021**;22(2):325-335. doi: 10.1007/s11154-020-09619-9.

Huang, C.K.; Shivaswamy, V.; Thaisetthawatkul, P.; Mack, L.; Stergiou, N.; Siu, K.C. An Altered Spatiotemporal Gait Adjustment during a Virtual Obstacle Crossing Task in Patients with Diabetic Peripheral Neuropathy. *J Diabetes Complications* **2019**, *33*, 182–188.

Paton J.S., Collings R., Glasser S., Kent B. The effects of foot and ankle devices on balance, gait and falls in adults with sensory perception loss: A systematic review protocol. [In Process] *JBI Library of Systematic Reviews* **2014,** 12:11 (74-91)

Shupert, C.L.; Horak, F.B. Adaptation of Postural Control in Normal and Pathologic Aging: Implications for Fall Prevention Programs. *J Appl Biomech* **1999**, *15*, 64–74.

No gait/balance (n = 7)

Heinen, A.; Lehmann, H.C.; Küry, P. Negative Regulators of Schwann Cell Differentiation - Novel Targets for Peripheral Nerve Therapies? *J Clin Immunol* **2013**, *33*.

Armstrong SA, Herr MJ. Physiology, Nociception. **2023** May 1. In: StatPearls [Internet]. Treasure Island (FL): StatPearls Publishing; 2023 Jan–. PMID: 31855389.

Baek, J.H.; Kim, H.; Oh, M.-K.; Park, K.-J.; Cho, Y.Y.; Kim, S.K.; Jung, J.H.; Kim, H.-J.; Jung, J.; Shin, H.S.; et al. Dynamic Postural Stability in Patients with Diabetic Peripheral Neuropathy and Relationship to Presence of Autonomic Neuropathy. **2018**, *23*, 303–311.

Chatwin, K.E.; Abbott, C.A.; Reddy, P.N.; Bowling, F.L.; Boulton, A.J.M.; Reeves, N.D. A Foreign Body Through the Shoe of a Person With Diabetic Peripheral Neuropathy Alters Contralateral Biomechanics: Captured Through Innovative Plantar Pressure Technology. *International Journal of Lower Extremity Wounds* **2018**, *17*, 125–129.

Gupta G., Maiya G.A., Bhat S.N., Hande H.M. Effect of Multifactorial Balance Rehabilitation Program on Risk of Falls and Functional Fitness in Older Adults with Diabetic Peripheral *Neuropathy Curr Aging Sci*. **2023**;16(3):240-247. doi: 10.2174/1874609816666230306150844.

Treister, R.; Lawal, O.D.; Shecter, J.D.; Khurana, N.; Bothmer, J.; Field, M.; Harte, S.E.; Kruger, G.H.; Katz, N.P. Accurate Pain Reporting Training Diminishes the Placebo Response: Results from a Randomised, Double-Blind, Crossover Trial. *PLoS One* **2018**, *13*.

van Baal, J.; Hubbard, R.; Game, F.; Jeffcoate, W. Mortality Associated with Acute Charcot Foot and Neuropathic Foot Ulceration. *Diabetes Care* **2010**, *33*, 1086–1089.

No VR/FB (n = 5)

Alaee, S.J.; Barati, K.; Hajiaghaei, B.; Ghomian, B.; Moradi, S.; Poorpirali, M. Immediate Effect of Textured Insoles on the Balance in Patients with Diabetic Neuropathy. *J Diabetes Investig* **2022**.

Beauchesne N, Wagenaar-Tison A, Brousseau-Foley M, Moisan G, Cantin V, Blanchette V. Using a contralateral shoe lift to reduce gait deterioration during an offloading fast walk setting in diabetic peripheral neuropathy: A comparative feasibility study. *Diabetes Res Clin Pract.* **2023**, 199:110647. doi: 10.1016/j.diabres.2023.110647.

du Plessis R., Dembskey N., Bassett S.H. Effects of an isometric exercise training program on muscular strength, ankle mobility, and balance in patients with diabetic peripheral neuropathy in the lower legs in South Africa. *Int J Diabetes Dev Ctries*. **2023**,43(2):252-257. doi: 10.1007/s13410-022-01068-1.

Ma OK, Ronsisvalle S, Basile L, Xiang AW, Tomasella C, Sipala F, Pappalardo M, Chan KH, Milardi D, Ng RC, Guccione S. Identification of a novel adiponectin receptor and opioid receptor dual acting agonist as a potential treatment for diabetic neuropathy. *Biomed Pharmacother*. **2023**, 158:114141. doi: 10.1016/j.biopha.2022.114141.

Prókai J, Murlasits Z, Bánhidi M, Csóka L, Gréci V, Atlasz T, Váczi M. The Effects of a 12-Week-Long Sand Exercise Training Program on Neuromechanical and Functional Parameters in Type II Diabetic Patients with Neuropathy. *Int J Environ Res Public Health*. **2023**, 20(7):5413. doi: 10.3390/ijerph20075413.

Contemporaneous FB/No pre-post (n = 1)

Naemi R., Healy A., Chockalingam N., Sundar L., Pillai A., Seeli_Abraham C., Snehalatha C., Ramachandran A. The contribution of visual feedback to balance in people with Type 2 diabetes and neuropathy. *Diabetic Medicine* **2013** 30 *SUPPL. 1* (70-71)

FULL-TEXT ARTICLES EXCLUDED (n = 10)

VR/FB only evaluative (n = 6)

Ahmad, I.; Noohu, M.M.; Verma, S.; Singla, D.; Hussain, M.E. Effect of Sensorimotor Training on Balance Measures and Proprioception among Middle and Older Age Adults with Diabetic Peripheral Neuropathy. *Gait Posture* **2019**, *74*, 114–120.

Abdul Razzak R., Hussein W. Postural visual dependence in asymptomatic type 2 diabetic patients without peripheral neuropathy during a postural challenging task. *J Diabetes Complications*. **2016**,30(3):501-6. doi: 10.1016/j.jdiacomp.2015.12.016.

Ahmed M.M., Mosalem D.M., Tarshouby W.A., Alfeeli A.K., Baqer A.B., Mohamed M.H. Computerized dynamic posturography in patients with diabetic peripheral neuropathy and visual feedback-based balance training effects (**2014**), 7 (2), pp. 267 – 272 DOI: 10.3889/MJMS.1857-5773.2014.0395

Huang C.-K., Siu K.-C., Shivaswamy V., Thaisetthawatkul P., Stergiou N. The Impact of Diabetic Peripheral Neuropathy on Patient's Balance Ability and Stepping Strategy – a Virtual Obstacle Crossing Study. *Archives of Physical Medicine and Rehabilitation* **2020**, 101:11 (e108-e109)

Najafi B., Bharara M., Talal T.K., Armstrong D.G. Advances in balance assessment and balance training for diabetes. *Diabetes Management* **2012** 2:4 (293-308)

Villegas C.M., Curinao J.L., Aqueveque D.C., Guerrero-Henríquez J., Matamala M.V. Identifying neuropathies through time series analysis of postural tests (**2023**), 99, pp. 24 – 34 DOI: 10.1016/j.gaitpost.2022.09.090

No gait/balance/risk of falling evaluation (n = 4)

De León Rodriguez, D.; Allet, L.; Golay, A.; Philippe, J.; Assal, J.P.; Hauert, C.A.; Pataky, Z. Biofeedback Can Reduce Foot Pressure to a Safe Level and without Causing New At-Risk Zones in Patients with Diabetes and Peripheral Neuropathy. *Diabetes Metab Res Rev* **2013**, *29*, 139–144.

Van Deursen, R.W.M.; Cavanagh, P.R.; Van Ingen Schenau, G.J.; Becker, M.B.; Ulbrecht, J.S. The Role of Cutaneous Information in a Contact Control Task of the Leg in Humans. *Hum Mov Sci* **1998**, *17*, 95–120.

Walker, S.C. Sensory Substitution in the Diabetic Neuropathic Foot. *J Am Podiatr Med Assoc* **1997**, *87*, 338.

York, R.M.; Perell-Gerson, K.L.; Barr, M.; Durham, J.; Roper, J.M. Motor Learning of a Gait Pattern to Reduce Forefoot Plantar Pressures in Individuals with Diabetic Peripheral Neuropathy. *PM and R* **2009**, *1*, 434–441.
